# TOR1 AIP1 interacts with p53 to enhance cell cycle dysregulation in prostate cancer progression

**DOI:** 10.1007/s11010-025-05276-1

**Published:** 2025-04-08

**Authors:** Zhaofeng Li, Xueyu Li, Han Yang, Meixiang Huang, Zhu Liu, Zongliang Zhang, Kai Zhao, Xinbao Yin, Guanqun Zhu, Yulian Zhang, Zhenlin Wang, Qinglei Wang, Zaiqing Jiang, Suofei Zhang, Tianzhen He, Ke Wang

**Affiliations:** 1 Department of Urology, The Affiliated Hospital of Qingdao University, Qingdao, Shandong China; 2 Hospital-Acquired Infection Control Department, Qingdao Central Hospital, Qingdao, Shandong China; 3 Lingzhushan Street Community Health Service Center (Lingzhushan Street Health Center), Huangdao District, Qingdao, China; 4 Department of Gynecology, The Affiliated Hospital of Qingdao University, Qingdao, Shandong China; 5 Department of Urology, Laixi People’s Hospital, Yantai, Shangdong China; 6 Institute of Special Environmental Medicine, Nantong University, Nantong, 226019 China

**Keywords:** TOR1 AIP1, Prostate adenocarcinoma, Proliferation, Invasion, Cell cycle dysregulation, P53

## Abstract

The cell cycle mechanism is an integration point where information is sent through an upstream signaling network, making it a potential target for cancer diagnosis and treatment. The LAP1 protein, encoded by the *Tor1aip1* gene, is required to maintain the shape of the nuclear envelope and the progression of the cell cycle. The aim of this study was to determine the role of *Tor1aip1* gene in PRAD development and its mechanism. We analyzed the expression and survival data of TOR1 AIP1 in PRAD patients in the TCGA database and verified the low expression of TOR1 AIP1 in prostate cancer by qPCR, western blot and immunohistology, which was correlated with the tumor stage and survival prognosis of PRAD. In addition, lentiviral vectors were used to mediate the up-regulation or down-regulation of TOR1 AIP1 expression in prostate cancer cells, and the effects of TOR1 AIP1 on tumor proliferation and related signaling pathways were investigated by cell counting kit- 8, colony formation assay, transwell assay, western blot, and flow cytometry. As a result, we found that TOR1 AIP1 enhances protein stability of p53 by directly interacting with p53, consequently inhibited tumor proliferation and invasion by inducing the cell cycle to be arrested in the S phase. Therefore, TOR1 AIP1 represents a promising therapeutic target in PRAD due to its ability to stabilize p53 and enhance its tumor-suppressive functions. Future studies should focus on elucidating its mechanisms, developing targeted therapies, and exploring its clinical potential in combination with existing treatments. By advancing our understanding of TOR1 AIP1, we may unlock new strategies for improving outcomes in PRAD patients.

**Supplementary Information:**

The online version contains supplementary material available at 10.1007/s11010-025-05276-1.

## Introduction

Prostate cancer (PRAD) is the most common malignant tumor in men [1], ranking first in incidence and second in mortality among Americans in 2020. There are expected to be 191,930 new cases, accounting for 21% of newly diagnosed cancers in men, and 33,330 deaths, accounting for 10% of cancer deaths [2]. In patients with low-intermediate risk localized PRAD, a wide range of treatment options are recommended, such as active surveillance, radical prostatectomy (RP), adjuvant external-beam radiation therapy (EBRT), and brachytherapy (BT) [3–5]. For those with advanced PRAD, RP with extended pelvic lymph node dissection and radiotherapeutic treatments (RT) with long-term androgen deprivation therapy (ADT) are strongly recommended [6]. However, not all patients are curable and 20–30% of these patients will develop castration-resistant prostate cancer (CRPC) in less than two years [7] after receiving ADT. The current limitations of PRAD treatment include ADT resistance [8], loss of p53 function [9], tumor heterogeneity [10], and limited response to immunotherapy [11]. Despite ongoing advancements, the precise mechanisms underlying these treatment challenges remain unclear, warranting further investigation.

Cell cycle dysregulation is a hallmark of prostate cancer, contributing to uncontrolled proliferation, genomic instability, and resistance to therapies [12]. The cell cycle is tightly regulated by cyclins, cyclin-dependent kinases (CDKs), and checkpoint proteins, including tumor suppressors, such as p53 and retinoblastoma protein (Rb). Alterations in these regulatory mechanisms are frequently observed in PRAD, promoting tumor growth and progression [13]. Loss of p53 function or Rb inactivation leads to the bypass of critical cell cycle checkpoints, allowing the proliferation of damaged or mutated cells [14]. Additionally, overexpression of cyclins (e.g., Cyclin D1) and hyperactivation of CDKs (e.g., CDK4/6) further drive tumor progression and contribute to treatment resistance, particularly in the development of castration-resistant prostate cancer (CRPC) [15]. The failure of androgen deprivation therapy (ADT), a standard treatment for advanced PRAD, is often associated with compensatory cell cycle dysregulation that supports tumor cell survival [16].

The protein coding gene Torsin 1 A-interacting protein 1 (*Tor1aip1*) encodes the LAP1 protein, which plays a crucial role in regulating cell cycle. LAP1 regulates nuclear membrane recombination [17–19] and mitotic spindle assembly in mitosis [20]. LAP1 can promote the assembly of the mitotic spindle by regulating nuclear envelope breakdown, microtubule dynamics, and cytoskeletal organization [21]. Diseases associated with TOR1 AIP1 include Myopathy, Autosomal Recessive, With Rigid Spine and Distal Joint Contractures, and Muscular Dystrophy [22]. TOR1 AIP1 was first shown to be associated with cancer in a report, claimed TOR1 AIP1 was determined to be one of the unique target genes of *p53* [23]. Compared with ovarian cancer, breast cancer stromal and normal stromal samples, Erceylan et al. identified the expression of HUB biomolecule TOR1 AIP1 in the tumor microenvironment gene network by systematic medical method and combined with survival analysis, confirmed that it is a prognostic marker in breast and ovarian tumor samples [24]. In a study of ulcerative colitis-associated colorectal cancer by Zhang et al., TOR1 AIP1 was identified as a crucial gene of UC-related carcinogenesis and functional enrichment analysis showed that it was related to mitochondrial dysfunction, cell–cell junction and immune response [25]. These findings suggest that TOR1 AIP1 plays a crucial role in the tumor progression, invasion, and metastasis. However, the underlying mechanism of TOR1 AIP1 in the tumorigenesis of PRAD remains unclear.

In this study, we analyzed TOR1 AIP1 expression and its association with the prognosis of PRAD patients in TCGA, LinkedOmics database. We also validated the expression of TOR1 AIP1 in the prostate cancer microenvironment by qPCR, western blot, and immunochemistry. In addition, we investigated the effect of TOR1 AIP1 on the development of PRAD. Consequently, TOR1 AIP1 interacted with p53 and consequently inhibited the viability, proliferation, and invasion of PRAD cell lines via dysregulating cell cycle. Our findings in this study firstly reported the important role of TOR1 AIP1 in prostate cancer.

## Materials and methods

### Data acquisition and processing

The RNA-Seq level 3 data and clinical data of prostate adenocarcinoma (PRAD) patients were downloaded from TCGA database (https://portal.gdc.cancer.gov/). A total of 553 cases were included in the study, consisting of 501 tumor patient groups and 52 normal adjacent groups. To identify the differentially expressed analysis and clinical relevance, we used deseq2 package (R version 3.6.3) for analysis and ggplot2 for data visualization (95% confidence interval: 0.048483–0.34348) [26]. To evaluate the differential expression of TOR1 AIP1, the Wilcoxon rank sum test was used.

### KM survival analysis

Survival analysis is a set of methods for evaluating time-to-event data that is widely applied across research disciplines. Standard survival analysis used the TCGA PRAD data and utilized the survminer and survival package to display the KM plots [27]. Using the “pROC” R program, the receiver operating characteristic (ROC) curve of diagnosis was produced (version 1.17.0.1).

### Human prostate sample collection

This study involved patients diagnosed with prostate cancer who underwent surgery at the Department of Urology, The Affiliated Hospital of Qindao University, between January 2022 and October 2023. Tissue samples were collected for experimental validation. This study complied with the principles set forth in the Helsinki Declaration and received approval from the Ethics Committee of Qindao University Hospital (Approval No. QYFYWZLL 26555, Qingdao, China). All participating patients and medical staff provided written informed consent. Both prostate cancer tissues and their corresponding normal prostate tissues were obtained from prostate specimens of patients who underwent radical prostatectomy. Patient information was shown in Table S1.Fresh surgery samples were obtained and divided into two parts: one part was sent to pathology for examination, and the other part was kept for qPCR, WB, and IHC. Prostate carcinoma tissue above 90% confirmed by pathology examination was used to detect the mRNA and protein expression level of TOR1 AIP1. All the specimens’ Gleason scores were 9. All Gleason scores were provided by six uropathologists of the Pathology Department of the Affiliated Hospital of Qingdao University.

### qRT-PCR

Following the manufacturer’s instructions, RNAiso Plus (Takara, Japan) was used to isolate total RNA from cell culture or tissue samples. Reverse transcription was carried out using the Evo M-MLV RT Kit with gDNA Clean for qPCR II on 1 g of total RNA (Accurate Biology, Hunan, China). Real-time PCR was performed using SYBR® Green Premix Pro Taq HS qPCR Kit (Accurate Biology, Hunan, China). For normalization, GAPDH acted as an endogenous control (Sangon Biotech, B661104). The following qRT-PCR primer sequences were displayed: human TOR1 AIP1 forward primer GGCTAGAAGAGTTCCGGTCC and reverse primer GGACACTGGTGGCTTCATATC.

### Western blot

We detected protein expression level of TOR1 AIP1 by WB. In detail, the total protein of specimens or cells was lysed with RIPA (Elabscience, Wuhan, China) containing 1 mM PMSF (Elabscience, Wuhan, China). Using a BCA protein assay kit (Elabscience, Wuhan, China), the protein concentration was determined. 60 μg of protein samples was separated by SDS-PAGE gel and transferred onto the PVDF membranes. After being blocked by 5% non-fat milk for two hours at room temperature, the membranes were probed with primary Abs [TOR1 AIP1 (Abcam, 1:5000), p53 (Abcam, 1:5000), CD1 (Servicebio, 1:500), CDK4 (Servicebio, 1:500), CDK6 (Servicebio, 1:500), and p21 (Absin, 1:1000)] overnight at 4 °C and then incubated with HRP-labeled secondary antibody for two hours at room temperature. The signal was visualized using Fusion Fx7 (VILBER LOURMAT).

### Immunohistochemistry

The tissue slides were incubated with primary antibody TOR1 AIP1 (E-AB- 52965, Elabscience, Wuhan, China) at 1:150 dilution overnight at 4 °C. The slices were then incubated with a horse radish peroxidase (HRP) antibody at a dose of 1:200 for 2 h at room temperature before being coated with 3,3-diaminobenzidine (DAB) (Servicebio, G1211). Light microscopy was used to observe all of the fields. The signals detected were specific, as evidenced by control studies conducted without primary antibodies.

### CO-IP

Co-IP analysis was performed with cells that stably expressed the target gene. The antibody is directly fixed to the agarose matrix using the Thermo Scientific™ Pierce™ Co-Immunoprecipitation (Co-IP) Kit. The whole-cell lysates were incubated with antibodies overnight at 4 °C and then precipitated with the antibody-protein complex. The immunoprecipitates were washed five times and then subjected to western blotting analysis.

### Cell lines and culture condition

Procell Life Science & Technology Co., Ltd was used to acquire the human prostate cancer cell lines PC3M, DU145, 22RV1 and C4 - 2 (Wuhan, China). These cells were grown in RPMI- 1640 culture medium and MEM culture medium, respectively, supplemented with 10% fetal bovine serum, 1% penicillin, and streptomycin. These cell lines were grown under a humidified atmosphere of 5% CO2 at 37 °C.

### Plasmid construction and cell transfection

For overexpression or knockdown of TOR1 AIP1, the lentivirus was chemically synthesized by OBiO Technology Company (Shanghai, China). PC3M cells, DU145 cells, 22RV1 cells and C4 - 2 cells were transfected with lentivirus plus 5-μg/ml polybrene for 16 h and the culture medium was replaced. Stable transformants of PC3M cells, DU145 cells, 22RV1 cells, and C4 - 2 cells were selected with 2-μg/ml puromycin or 8-μg/ml blasticidin (MedChemExpress, Shanghai, China) for more than 7 days.

### Cell proliferation and colony formation assays.

A cell counting kit- 8 was used to examine cell growth (CCK8; MedChemExpress, Shanghai, China). 1000 cells/well were used to seed the cells in 96-well plates. 10 μl CCK8 reagent was added to 100-μl culture medium in each well and then incubated at 37 °C for 2 h. The absorbance values were detected using a microplate reader at 450 nm. The colony formation assay was performed at a density of 1000 cells/well in a 6-well plate. After one week of culture, cells were fixed with 4% paraformaldehyde for 30 min at room temperature and washed two times with PBS. The cells were stained with 0.5% crystal violet for 30 min and images were taken following a twice wash of PBS.

### Cell migration and invasion assay

The Transwell chambers (pore size: 8 μm, Corning) with Matrigel-coated surfaces were used for the cell invasion assay. The matrix glue was diluted with the serum-free medium at 1:5. There were 500 μl of 10% FBS medium in the lower chamber. Transfected tumor cells (5 × 10^4^) in 200 μl of serum-free medium were gently loaded onto the upper chamber and incubated at 37 °C for 24 h. The filter inserts were taken out of the chambers and stained with 0.1% crystal violet after being fixed in 600 μl of 4% paraformaldehyde for 30 min. Cotton swabs were used to remove the cells from the top side of the membrane and an inverted microscope was used to see the invaded cells (Olympus, Tokyo, Japan). All results were repeated three times.

### Cell cycle analysis

Cell cycle analysis of control and TOR1 AIP1-overexpressed DU145 and PC3M cells from 3 independent biological replicates were collected. These cells were suspended with 1 ml 75% ethanol precooled at − 20 °C and stayed overnight at 4 °C. The fixed cells were rinsed with PBS and resuspended with 100ul PBS containing 10-mg/ml RNase A (Servicebio, G3405 - 1ML) for 30 min at 37 °C, then incubated with 100-ul 100-ug/ml propidium iodide (PI) (Servicebio, G1021) for 10 min in the dark. Finally, the mixture was analyzed using CytoFLEX flow cytometry (Beckman, USA).

### Xenotransplantation mouse model

In vivo experiments, 2 × 10^7^ PRAD cell suspensions were mixed with matrix gel 2:1 and injected into the underarm of 6-week-old male BALB/c-nu thymus nude mice (purchased from Jinan Pengyue Experimental Animal Breeding Co., Jinan, China; 6 mice per group). Subcutaneous tumor formation was observed from the 2nd week after injection, and tumor size was measured weekly with Vernier calipers. The calculation formula of tumor volume is as follows: (length × width^2^)/2. At the end of the experiment, the mice were euthanized, and the tumor tissues were weighed. The tumor from mice was taken for immunohistochemistry and western blot.

### Statistical analysis

For bioinformatic analysis, all statistical analyses were performed using R language (version 3.6.3). For bioassay validation, comparisons of two groups of data were analyzed by a two-tailed Student’s *t* test using GraphPad Prism 7.0. (GraphPad, San Diego, CA). For comparisons involving more than two groups, one-way ANOVA was performed. All P values less than 0.05 were considered significant.

## Results

### The expression of TOR1 AIP1 was downregulated in prostate cancer specimens

**Fig. 1:**
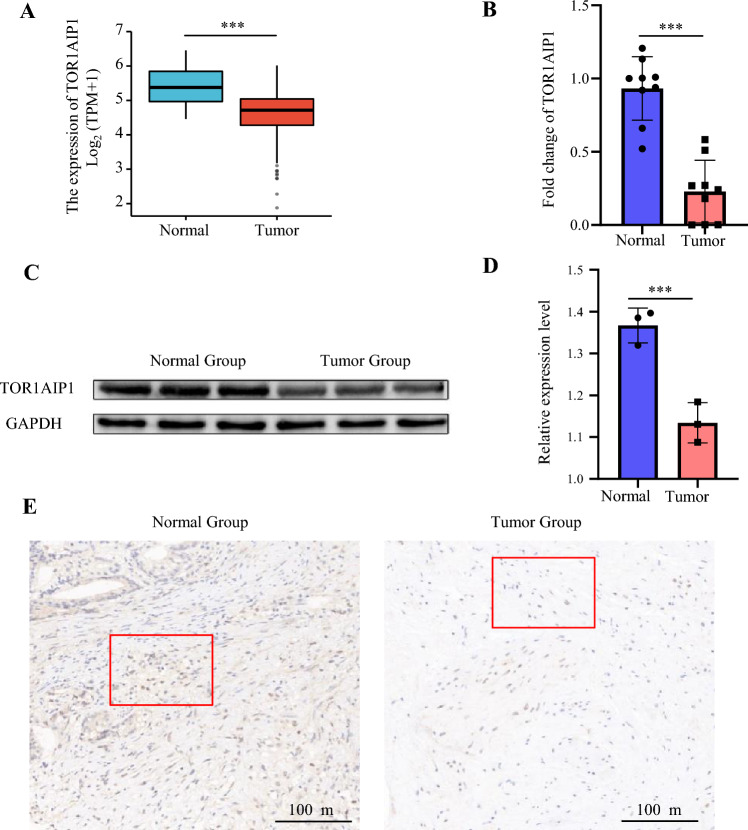
The expression of TOR1 AIP1 was downregulated in prostate cancer samples. **A** The expression of TOR1 AIP1 in 499 tumor tissues and 52 normal tissues was analyzed by TCGA. **B** The expression level of TOR1 AIP1 was validated by qPCR using clinical samples (*n* = 5). **C** The protein level of TOR1 AIP1 in human prostate tumor specimens was determined by WB (*n* = 3). **D** The expression of TOR1 AIP1 in prostate cancer was examined by IHC (*n* = 3). Normal group: Non-cancerous tissue adjacent to the tumor, Tumor group: neoplastic tissue. Compared with the indicated group, *P < 0.05, **P < 0.01, and ***P < 0.001

We analyzed the expression of TOR1 AIP1 in 52 normal prostate samples and 499 prostate adenocarcinoma (PRAD) samples from The Cancer Genome Atlas in order to determine the role that this gene plays in the development of PRAD (TCGA). The findings indicated that patients with prostate cancer had lower levels of TOR1 AIP1 expression (Fig. 1A).We obtained both prostate cancer tissues and normal prostate tissues from the prostate specimens of five prostate cancer patients. All five prostate cancer patients had adenocarcinoma with a Gleason score of 9, including three at tumor stage T2NxMx and the other two at T3bNxMx (Table S1). Real-time PCR was performed to confirm the TOR1 AIP1 mRNA expression level in PRAD and normal tissues, and it was shown that TOR1 AIP1 was considerably downregulated in PRAD samples compared to normal samples (Fig. 1B). Then, using Western blot and immunohistology, we confirmed the protein level of TOR1 AIP1 in both tumor and normal prostate samples. Similarly, TOR1 AIP1 expression was decreased in tumor tissues compared to healthy samples (Figs. 1C–E, S1).

**Fig. 2:**
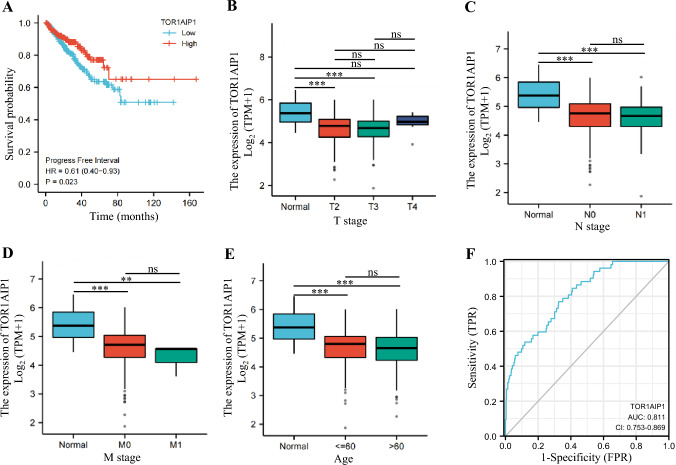
The prognostic value of TOR1 AIP1 in prostate cancer patients. **A** The prognostic value of mRNA Level of TOR1 AIP1 in prostate cancer patients (Kaplan–Meier Plotter). **B**–**E** Lower TOR1 AIP1 expression was associated with age, N stage, T stage, and M stage. **F** ROC curve analysis of TOR1 AIP1. Compared with the indicated group, *P < 0.05, **P < 0.01, and ***P < 0.001

In addition, we investigated the Kaplan–Meier plotter for the prognostic significance of TOR1 AIP1 using the survminer and survival package of the R project. The samples were divided into high expression group and low expression group by median of TOR1 AIP1 expression. As a result, it showed that patients with low TOR1 AIP1 expression have significantly shorter PFS than patients with high TOR1 AIP1 levels (Fig. 2A). Then, we further investigated the correlation between the expression of TOR1 AIP1 and clinical parameters in PRAD. The clinical features of PRAD, such as age, T stage and M stage, were related to the levels of TOR1 AIP1 (Fig. 2B–E). Additionally, the AUC value for TOR1 AIP1 was 0.811, regarded as appropriate for prediction in PRAD (Fig. 2F).

### TOR1 AIP1 overexpression inhibited the proliferation, migration, and invasion of PRAD cells in vitro

**Fig. 3:**
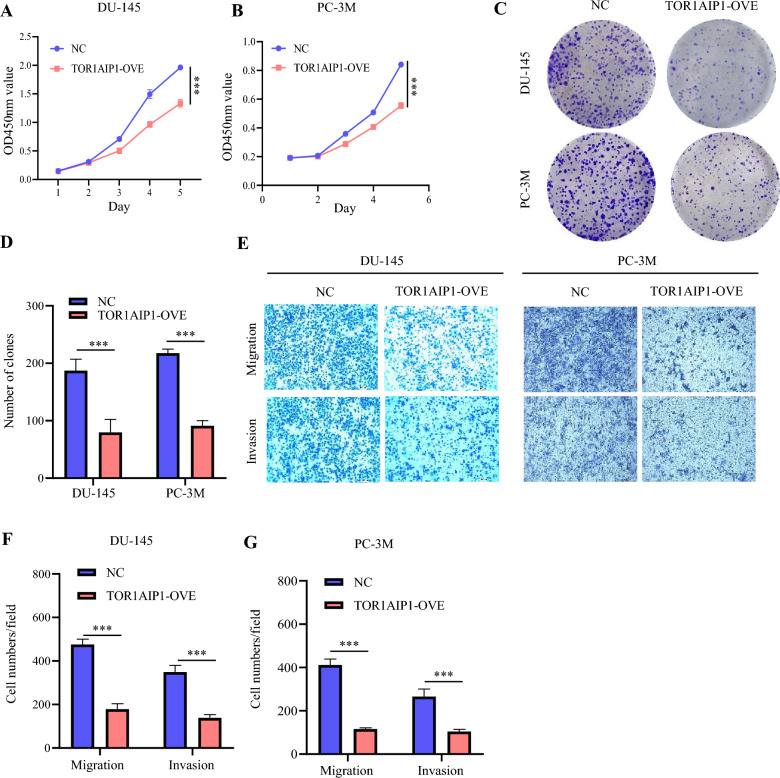
TOR1 AIP1 inhibited the proliferation and invasion of the DU145 and PC3M cells. TOR1 AIP1 stably overexpression DU145 cells and PC3M cells using lentivirus were generated. **A** and **B** The viability of DU145 and PC3M cells were measured by CCK8 assay at the indicated times. **C** and **D** Colony formation assays were performed in DU145 cells and PC3M cells transfected with indicated lentivirus. **E**–**G** The penetrability in Transwell chambers was measured to verify the invasive capability of DU145 cells and PC3M cells. Data represent mean ± SD (*n* = 3). NC: Control virus-transfected cells, TOR1 AIP1 OVE: TOR1 AIP1-overexpressing cells. Compared with the indicated group, *P < 0.05, **P < 0.01, and ***P < 0.001

To better understand the biological effect of TOR1 AIP1 in PRAD cells in vitro, we determined the expression of TOR1 AIP1 in the normal prostate epithelial cell line RWPE- 1 and five types of prostate cancer cell lines, including 22RV1, C4 - 2, C4 - 2B, DU145, and PC3M by western blotting (Fig. S2). We found that TOR1 AIP1 was downregulated in prostate cancer cells compared with RWPE- 1 cells. In addition, the expression levels of TOR1 AIP1 in 22RV1 and C4 - 2 cell lines were markedly higher than in other prostate cancer cell lines. In contrast, the expression levels of TOR1 AIP1 in DU145 and PC3M cells were markedly lower than in other prostate cancer cell lines. Hence, we employed lentivirus to increase the expression of TOR1 AIP1 in DU145 cells and PC3M cells. The overexpression efficacy of TOR1 AIP1 was shown in Figure S3. TOR1 AIP1 overexpression in DU145 cells and PC3M cells inhibited proliferation ability markedly via CCK8 assay (Fig. 3A, B), and colony formation assay (Fig. 3C, D). In addition, TOR1 AIP1 overexpression in DU145 cells and PC3M cells also suppressed migrative and invasive ability via transwell migration and invasion assay (Fig. 3E–G).

**Fig. 4:**
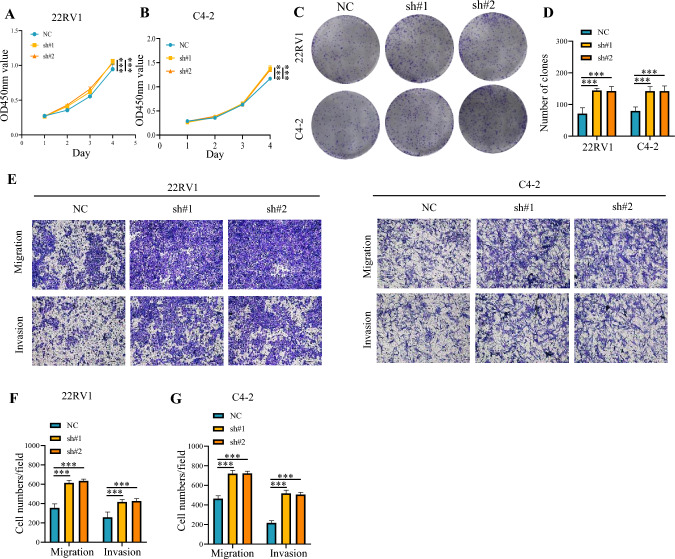
shRNA-mediated silencing of TOR1 AIP1 increased the proliferation, migration, and invasion of PRAD cells. TOR1 AIP1 stably knockdown 22RV1 and C4 - 2 cell lines using lentivirus were generated. **A** and **B** The viability of 22RV1 and C4 - 2 cells were measured by CCK8 assay at the indicated times. **C** and **D** Colony formation assays were performed in 22RV1 and C4 - 2 cells transfected with indicated lentivirus. **E**–**G** Transwell assays were used to detect the migration and invasion ability of 22RV1 and C4 - 2 cells. NC: Control virus-transfected cells, sh#1 and sh#2: TOR1 AIP1 knockdown cells. Data represent mean ± SD (*n* = 3). By comparison with vehicle control group, *P < 0.05 and ***P < 0.001

Furthermore, we generated TOR1 AIP1 stably knockdown 22RV1 and C4 - 2 cell lines using lentivirus. The knockdown efficacy of TOR1 AIP1 was determined by western blot (Fig. S4). Subsequently, CCK8 and colony formation experiments validated that the knockdown of TOR1 AIP1 was essential for promoting the proliferation of prostate cancer cells (Fig. 4A–D). In addition, the transwell assay also showed that knockdown of TOR1 AIP1 markedly increased the migration and invasion abilities of 22RV1 and C4 - 2 cells (Fig. 4E–G).

### Overexpression of TOR1 AIP1 inhibited the progression of PRAD in vivo

**Fig. 5:**
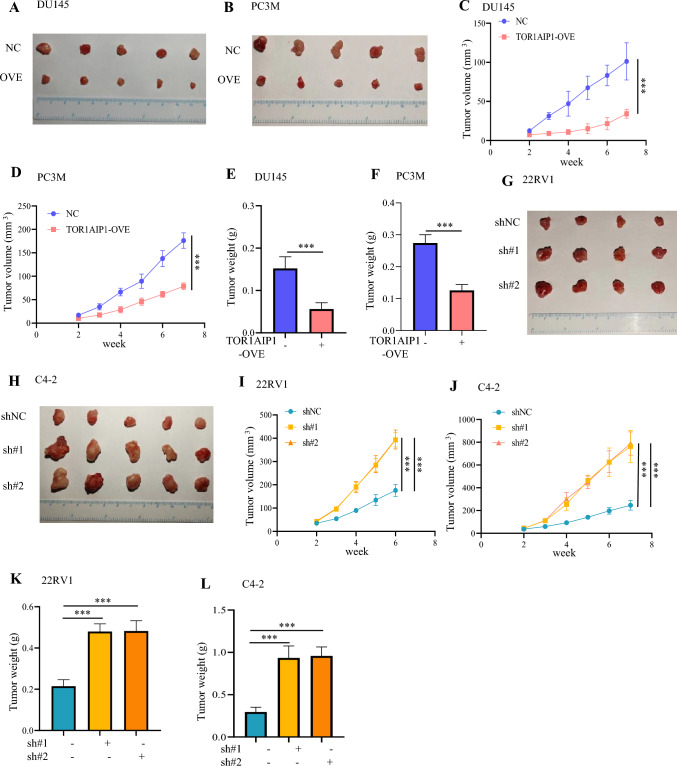
TOR1 AIP1 overexpression suppressed the progression of PRAD in vivo. We generated TOR1 AIP1 stably overexpressed DU145 cells and PC3M cells (TOR1 AIP1 OVE) or TOR1 AIP1 stably knockdown 22RV1 cells and C4 - 2 cells (sh#1 and sh#2) and control cells (shNC) and injected them subcutaneously into the right axils of nude mice. **A** and **B** Xenograft tumor images of DU145 cells and PC3M cells stably transfected with NC or TOR1 AIP1 overexpression. **C** and **D** Tumor Weight of DU145 cells and PC3M cells stably transfected with NC or TOR1 AIP1 overexpression (*n* = 5). **E** and **F** The growth curve of DU145 cells and PC3M cells stably transfected with NC or TOR1 AIP1 overexpression in nude mice (*n* = 5). **G** and **H** Xenograft tumor images of 22RV1 cells and C4 - 2 cells stably transfected with shNC or TOR1 AIP1 sh#1 and sh#2 (*n* = 4). **I** and **J** Tumor weight of shNC and TOR1 AIP1 sh#1 and sh#2 (*n* = 4). **K** and **L** The growth curve of different groups (*n* = 4). NC and shNC: Nude mice inoculated with control virus-transfected cells, OVE: Nude mice injected with TOR1 AIP1-overexpressing cells, sh#1 and sh#2: Nude mice injected with TOR1 AIP1 knockdown cells. Data represent mean ± SD. Compared with the indicated group, ***P < 0.001

We next established a nude mouse model to further understand the in vivo role of TOR1 AIP1 in PRAD progression. And we generated TOR1 AIP1 stably overexpressed cells (TOR1 AIP1 OVE) and control cells (NC) and injected them subcutaneously into the right axils of nude mice. The results showed that TOR1 AIP1 overexpression suppressed tumor growth, with significantly smaller tumor volumes and tumor weights than controls (*P* < 0.001, Fig. 5A–F). The immunohistochemical and western blotting results showed that overexpression of TOR1 AIP1 decreased the expression level of proliferation marker Ki67, whereas increased the expression level of p53 protein (Fig. S5). Taken together, the above data confirmed the anti-tumor role of TOR1 AIP1 in the progression of PRAD.

In addition, we injected TOR1 AIP1 stably knockdown cells (sh#1 and sh#2) and control cells (shNC) subcutaneously into the right axilla of nude mice. The results showed that TOR1 AIP1 knockdown markedly promoted tumor growth, with significantly bigger tumor volumes and tumor weights than controls (*P* < 0.001, Fig. 5G–L).

### TOR1 AIP1 inhibits the progression of PRAD by dysregulating cell cycle

**Fig. 6:**
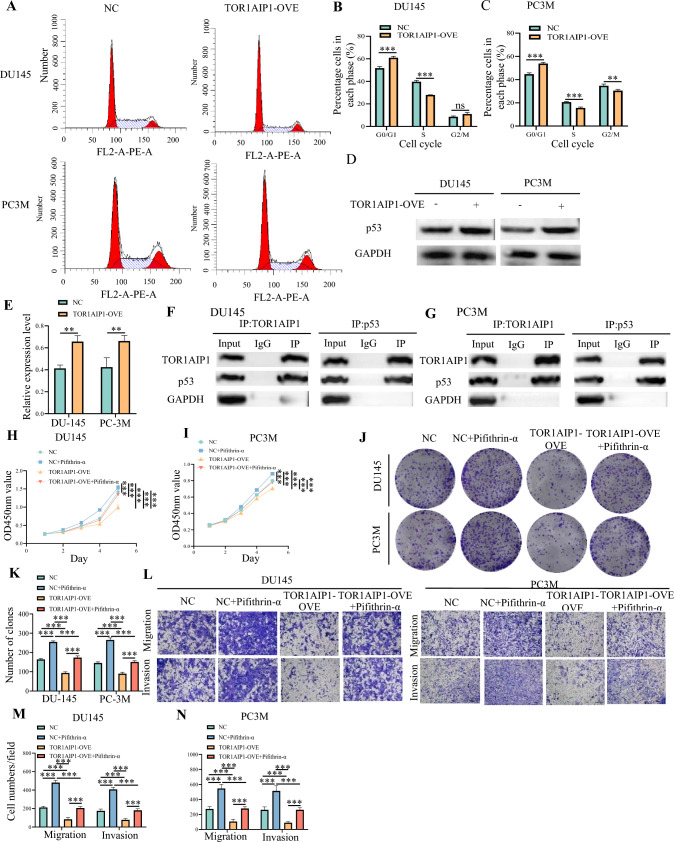
TOR1 AIP1 interacts with p53 to inhibit the tumor progression in vitro. **A**–**C** Cell cycle analysis was performed using flow cytometry. **D** and **E** The expressions of p53 protein in DU145 and PC3M cells with stably transfected with NC or TOR1 AIP1 overexpression were determined by western blot. **F** and **G** The interaction between TOR1 AIP1 and p53 in DU145 and PC3M cells was determined by CO-IP. **H** and **I** The viability of TOR1 AIP1 stably overexpression DU145 cells and PC3M cells with or without pifithrin-α treatment were measured by CCK8 assay. **J** and **K** Colony formation assays were performed in TOR1 AIP1 stably overexpression DU145 cells and PC3M cells with or without pifithrin-α treatment. **L**–**N** Transwell assay was used to detect the effect of pifithrin-α on migration and invasion of TOR1 AIP1 stably overexpression DU145 cells and PC3M cells. Data represent mean ± SD (*n* = 3). NC: Control virus-transfected cells, TOR1 AIP1 OVE: TOR1 AIP1-overexpressing cells. Compared with the indicated group, ***P < 0.001, n.s, no significant difference

To comprehend the underlying mechanism of the effect of TOR1 AIP1 on PRAD cells, we determined the cell cycle of PRAD after TOR1 AIP1 overexpression. As a result, overexpression of TOR1 AIP1 markedly increased the ratios of the G0/G1 phase and inhibited the ratios of the S phase (Fig. 6A–C). These results suggested that TOR1 AIP1 overexpression inhibited PRAD proliferation and invasion via regulating the cell cycle.

### TOR1 AIP1 interacts with p53 to inhibit the tumor progression

To further determine which cell cycle signaling pathway(s) are required for the proliferation and invasion of PRAD cells, we determined the expressions of Cyclin D1, CDK4, CDK6, p21 (Fig. S6), and p53 proteins of TOR1 AIP1 stably overexpressed cells by western blotting assays. The results showed that the expression of p53 protein in TOR1 AIP1 stably overexpressed cells was significantly increased compared with control cells (Figs. 6D, E, S7). In contrast, p53 mRNA levels remained unchanged in cells that were stably overexpressed with TOR1 AIP1, in contrast to the control cells (Fig. S8). Hence, TOR1 AIP1 promoted the protein stability of p53. TOR1 AIP1 may affect the ubiquitination-mediated degradation of p53.

Moreover, as shown in Fig. 6F and G (Fig. S9), we found a TOR1 AIP1 directly interacted with p53 in both DU145 and PC3M cell lines. To further confirm that the p53 expression might serve as the molecular mechanism of TOR1 AIP1 involved in the development of PRAD, we used pifithrin-α (MedChemExpress, Shanghai, China), an inhibitor of p53 protein, to determine if it resulted in rescuing the effect of TOR1 AIP1 on tumor progression. The TOR1 AIP1 stably overexpressed cells were treated with 15-μM Pifthrin-α for 24 h and then collected for further experiments. By CCK8 (Fig. 6H, I), colony formation assay (Fig. 6J, K), and transwell assay (Fig. 6L–N), pifthrin-α was shown to rescue TOR1 AIP1-mediated anti-tumor effect in human PRAD cell lines.

**Fig. 7:**
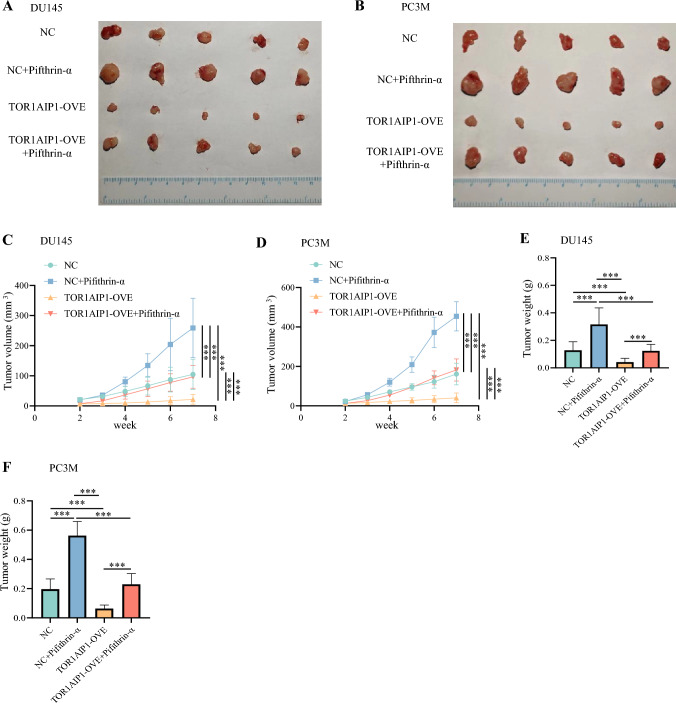
p53-mediated the anti-tumor effect of TOR1 AIP1 in vivo. We injected subcutaneously TOR1 AIP1 stably overexpression cells, with or without pifithrin-α intratumoral treatment for nude mice. **A**–**F** Tumor images (**A**, **B**), tumor weights (**C**, **D**), and tumor growth curve (**E**, **F**) of different groups. Data represent mean ± SD (*n* = 5). NC: Nude mice inoculated with control virus-transfected cells, OVE: Nude mice injected with TOR1 AIP1-overexpressing cells. Compared with the indicated group, **P < 0.01 and ***P < 0.001

Furthermore, to explore whether p53 mediated the anti-tumor effect of TOR1 AIP1 in vivo, we injected subcutaneously TOR1 AIP1 stably overexpression cells, with or without pifthrin-α intratumoral treatment for nude mice. As shown in Fig. 7A–F, pifthrin-α almost completely rescued the inhibitory effects of TOR1 AIP1 overexpression on mouse tumor growth. Collectively, TOR1 AIP1 interacted with p53 to enhance cell cycle dysregulation, which was crucial for the anti-tumor role of TOR1 AIP1 in the progression of PRAD in vitro and in vivo.

## Discussion

Prostate cancer is one of the world’s most common male genitourinary tumors [28]. Standard therapies for localized illness include radical prostatectomy and radiotherapy, both of which offer good cancer control. However, approximately 20–35% of treated patients develop a biochemical recurrence (BCR) [29, 30]. For patients undergoing BCR or initially presenting with metastatic cancer, androgen deprivation therapy is the first line of treatment for these patients [31, 32]. Unfortunately, after an initial clinical response, this disease will eventually develop into castration-resistant prostate cancer (CRPC) [33, 34], which still represents a poor prognosis. Therefore, developing a novel prognostic marker for prostate cancer is necessary. In this study, we identified TOR1 AIP1 as a novel potential prognostic marker for prostate cancer and further investigated the correlation between TOR1 AIP1, cellular proliferation and invasion, and gene mutation in PRAD.

Torsin 1 A-interacting protein 1 (TOR1 AIP1), also known as LAP1, LAP1B, LAP1 C, and LGMD2Y, encodes LAP1 protein, which is one of the earliest laminin-related proteins [35]. In this study, we analyzed the expression and survival data of TOR1 AIP1 in PRAD patients from TCGA, LinkedOmics, and the TISIDB database. We found that the expression of TOR1 AIP1 was markedly downregulated in PRAD compared to the normal samples. Furthermore, the expression of TOR1 AIP1 was associated with advanced tumor stage and survival prognosis. Survival analysis showed that low expression of TOR1 AIP1 was correlated with PFS. We also validated the TOR1 AIP1 mRNA expression in human prostate tumor specimens by qPCR. In consistent with the predicted results by bioinformatic analysis, the mRNA level of TOR1 AIP1 was markedly decreased in the PRAD group. In addition, we investigated the protein level of TOR1 AIP1 in prostate cancer by Western blot and IHC. These results indicated that TOR1 AIP1 was a prognostic biomarker for PRAD. The failure of androgen deprivation therapy, a standard treatment for advanced PRAD, is often associated with compensatory cell cycle dysregulation that supports tumor cell survival [16]. In our study, we validated that TOR1 AIP1 serves as a promising biomarker for prostate cancer treatment. Further investigation should focus on determining how TOR1 AIP1 synergizes with androgen deprivation therapy.

To better comprehend the underlying mechanism of the effect of TOR1 AIP1 on PRAD cells, we determined the cell cycle of PRAD after TOR1 AIP1 overexpression. As a result, overexpression of TOR1 AIP1 markedly increased the ratios of the G0/G1 phase and inhibited the ratios of the S phase. These results suggested that TOR1 AIP1 overexpression inhibited PRAD proliferation and invasion via regulating the cell cycle. Furthermore, we determined the expressions of CD1, CDK4, CDK6, p53, and p21 proteins of TOR1 AIP1 stably overexpressed cells by western blotting assays. The results showed that the expression of p53 protein in TOR1 AIP1 stably overexpressed cells was significantly increased. Meanwhile, the p53 inhibitor Pifithrin-α was shown to rescue the TOR1 AIP1-mediated anti-tumor effect in human PRAD cell lines. Considering the limitations of p53 inhibitors, such as off-target effects and limited inhibition of p53 functions [36], additional p53 inhibitors or genetic p53 knockdown should be used for validation in future studies. The expression of p53 mRNA in TOR1 AIP1 stable overexpressed cells was detected by qPCR. The results showed that there was no significant difference in the expression of p53 mRNA in TOR1 AIP1 stably overexpressed cells. Therefore, we concluded that TOR1 AIP1 can promote the stability of p53 protein. However, the underlying mechanism of the increasing p53 expression in TOR1 AIP1 stably overexpressed cells needs to be further studied in future. Furthermore, we also showed that TOR1 AIP1 interacted with p53, consequently inhibited tumor proliferation and invasion by inducing the cell cycle to be arrested in the S phase. Our findings were strengthened by previous studies. In acute myeloid leukemia, Kojima et al. [37] showed that inhibiting CDK1 increased p53-mediated mitochondrial apoptosis via Bax activation and the G2/M phase cell cycle arrest. Chen et al. provided evidence supporting the notion that the activation of p53 and p21 may effectively suppress the expression of CDKs, E2 Fs, and other factors that facilitate DNA replication during the G1/S phase arrest in bladder cancer [38]. In a study conducted by Danupon et al. it was shown that the CCNB1/CDK1 complex underwent translocation to the mitochondria during the G2/M phase. This translocation facilitated the phosphorylation and subsequent activation of p53 at Ser- 315, hence triggering an anti-apoptotic response in HCT116 cells 0.28 [39]. According to Bowen et al. it was showed that the regulation of mitochondrial energy metabolism by CCNB1/CDK1 had a role in promoting cell cycle progression and enhancing the tumor’s response to radiation [40, 41]. The occurrence of abnormal mitosis, which is triggered by the CCNB1/CDK1 complex, plays a significant role in the genesis and progression of cancer [42]. Previously, TOR1 AIP1 may serve as the target gene of *p53* [23]. However, our study first confirmed that TOR1 AIP1 interacted with p53 and increased p53 protein expression, without altering the mRNA level of p53. This might be attributed to the recruitment of deubiquitylating enzymes, such as USP7, by TOR1 AIP1, resulting in an elevation of p53 protein expression and stability [43]. Further study should investigate whether TOR1 AIP1 affect the ubiquitination-mediated degradation of p53. We thus conclude that the axis of TOR1 AIP1-p53 suppressed tumor growth via inducing the cell cycle to be arrested in the S phase. The TOR1 AIP1-p53 axis has great potential for translational applications in the treatment of PRAD, including enhancing p53 stability, combining with existing therapies, developing new targeted drugs, gene therapy, and cell therapy. Despite challenges such as biomarker validation, drug delivery, and clinical trial design, the TOR1 AIP1-p53 axis offers new therapeutic strategies for PRAD patients, with the potential to significantly improve prognosis. Future research should focus on these translational applications to facilitate their rapid transition from the laboratory to the clinic.

All in all, our study found that the expression of TOR1 AIP1 is remarkably downregulated in PRAD specimens compared with the normal samples. The expression of TOR1 AIP1 was correlated with advanced tumor stage and survival prognosis in PRAD. Consistent with the results of our computational analysis, TOR1 AIP1 was downregulated in PRAD specimens. Furthermore, we found that TOR1 AIP1 promoted the protein stability of p53 by directly interacting with p53, consequently inhibited tumor proliferation and invasion by inducing the cell cycle to be arrested in the S phase. In this study, we have, for the first time, demonstrated the critical role of TOR1 AIP1 in prostate cancer progression, specifically its mechanism of regulating cell cycle dysregulation through interaction with p53 to suppress tumor development. Based on these findings, we propose that TOR1 AIP1 can serve as a diagnostic biomarker and prognostic indicator for prostate cancer. Furthermore, the discovery of the TOR1 AIP1-p53 interaction provides a novel target for personalized therapy in prostate cancer and opens new avenues for the development of targeted drugs. The underlying mechanism of TOR1 AIP1 promoting the stability of p53 needs further investigation in future experiments.

## Supplementary Information

Below is the link to the electronic supplementary material.

Supplementary file1 (DOCX 3880 KB)

## Data Availability

RNA-seq data can be obtained from TCGA (https://portal.gdc.cancer.gov/) PRAD. Data of this study are available upon request from the authors.

## Abbreviations

PRAD: Prostate cancer
CRPC: Castration-resistant prostate cancer
TOR1 AIP1: Torsin 1 A-interacting protein 1
LAP1: Lamina-associated polypeptide 1
PFS: Progression-free survival
BCR: Biochemical recurrence
CDK: Cyclin-dependent kinases
RP: Radical prostatectomy
EBRT: External beam radiation therapy
BT: Brachytherapy
RT: Radiotherapeutic treatments
ADT: Androgen deprivation therapy

## Declarations

### Research involving animal participants

All animal study protocols were approved by the Ethics Committee of the Medical Animal Laboratory of the Affiliated Hospital of Qingdao University (No: AHQU-MAL20230512).
