# Supplementary material for: TOR1 AIP1 interacts with p53 to enhance cell cycle dysregulation in prostate cancer progression
Source: Mol Cell Biochem. 2025 Apr 8;480(7):4483–97. doi: 10.1007/s11010-025-05276-1 (PMC12263815; doi:10.1007/s11010-025-05276-1)
Supplement: Supplementary file 1 — Supplementary file1 (DOCX 3880 KB) [file 11010_2025_5276_MOESM1_ESM.docx]

***Supplementary information***

**TOR1AIP1 interacts with p53 to enhance cell cycle dysregulation in prostate cancer progression**

**Zhaofeng Li^1†^, Xueyu Li^1,2†^, Han Yang^1^, Meixiang Huang^2^, Zhu Liu^3^, Zongliang Zhang^1^, Kai Zhao^1^, Xinbao Yin^1^, Guanqun Zhu^1^, Yulian Zhang^4^, Zhenlin Wang^1^, Qinglei Wang^1^, Zaiqing Jiang^1^, Suofei Zhang^5^, Tianzhen He^6*^, Ke Wang^1*^**

1 Department of Urology, The Affiliated Hospital of Qingdao University, Qingdao, Shandong, China

2 Hospital-Acquired Infection Control Department, Qingdao Central Hospital, Qingdao, Shandong, China

3 Lingzhushan Street Community Health Service Center (Lingzhushan Street Health Center), Huangdao District, Qingdao, China

4 Department of Gynecology, The Affiliated Hospital of Qingdao University, Qingdao, Shandong, China

5 Department of Urology, Laixi People's Hospital, Yantai, Shangdong, China

6 Institute of Special Environmental Medicine, Nantong University, Nantong 226019, China

† These authors contributed equally to this work and share first authorship.

*Correspondence

Tianzhen He E-mail: [sailing198562@ntu.edu.cn](mailto:sailing198562@ntu.edu.cn)

Ke wang E-mail: wangke@qdu.edu.cn

**Supplementary Figures**

**Figure S1**


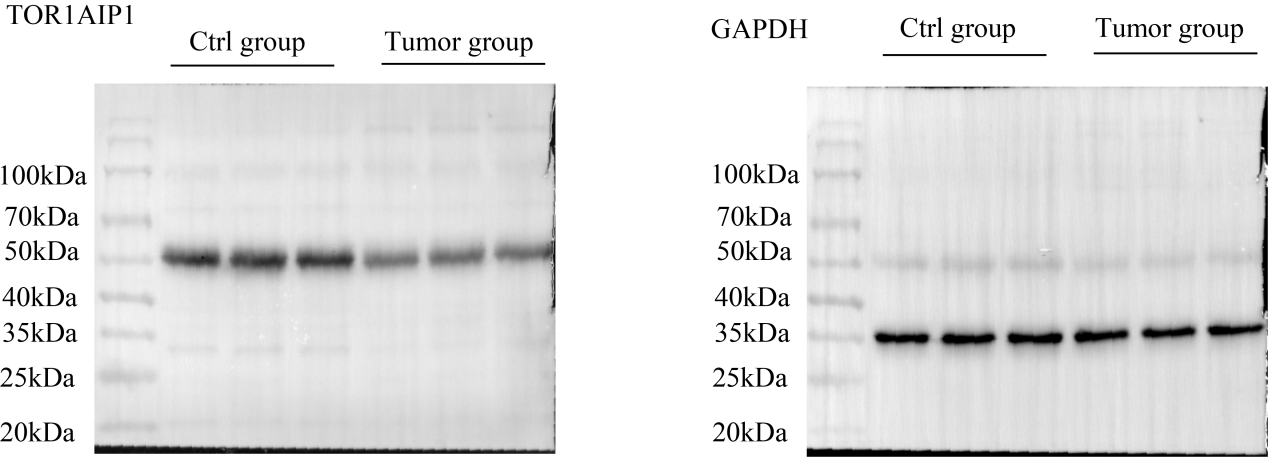


**Raw data of western blot for Fig 1C.**

**Figure S2**


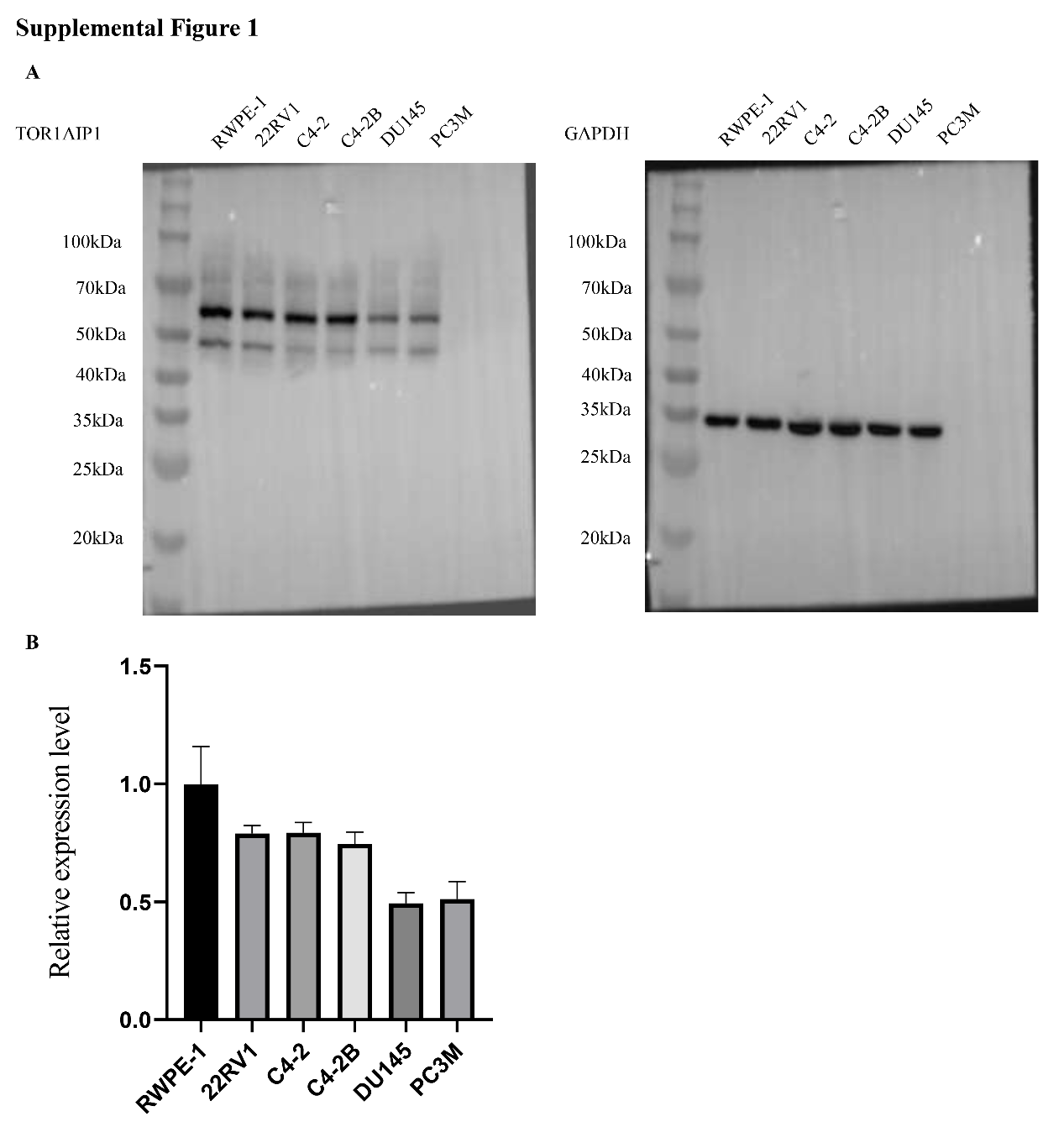


**The protein levels of TOR1AIP1 in the normal prostate epithelial cell line RWPE-1 and five types of prostate cancer cell lines.**

**Figure S3**


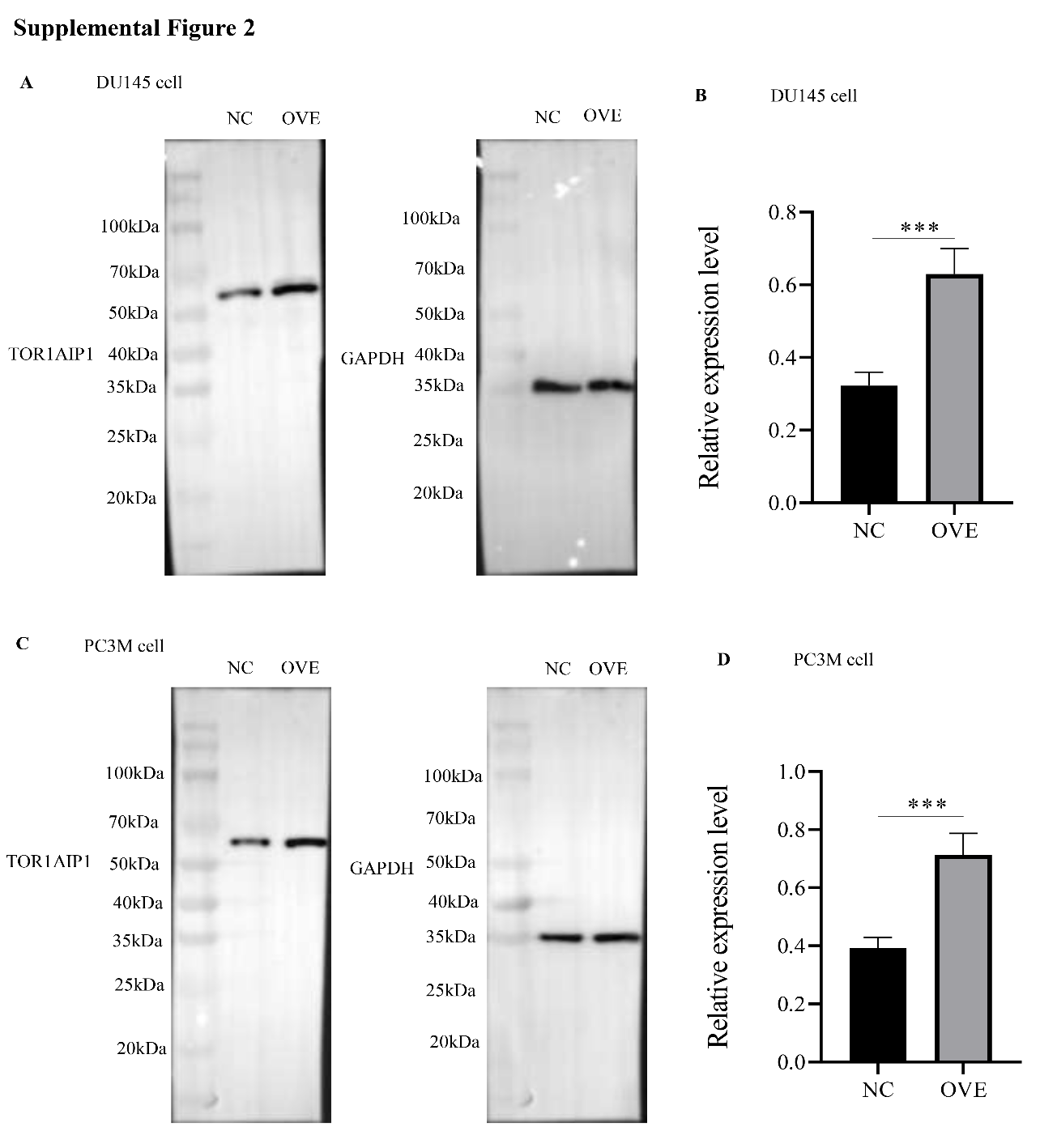


**The overexpression efficacy of TOR1AIP1. (A-D)** The overexpression efficacy of TOR1AIP1was determined by WB.

**Figure S4**


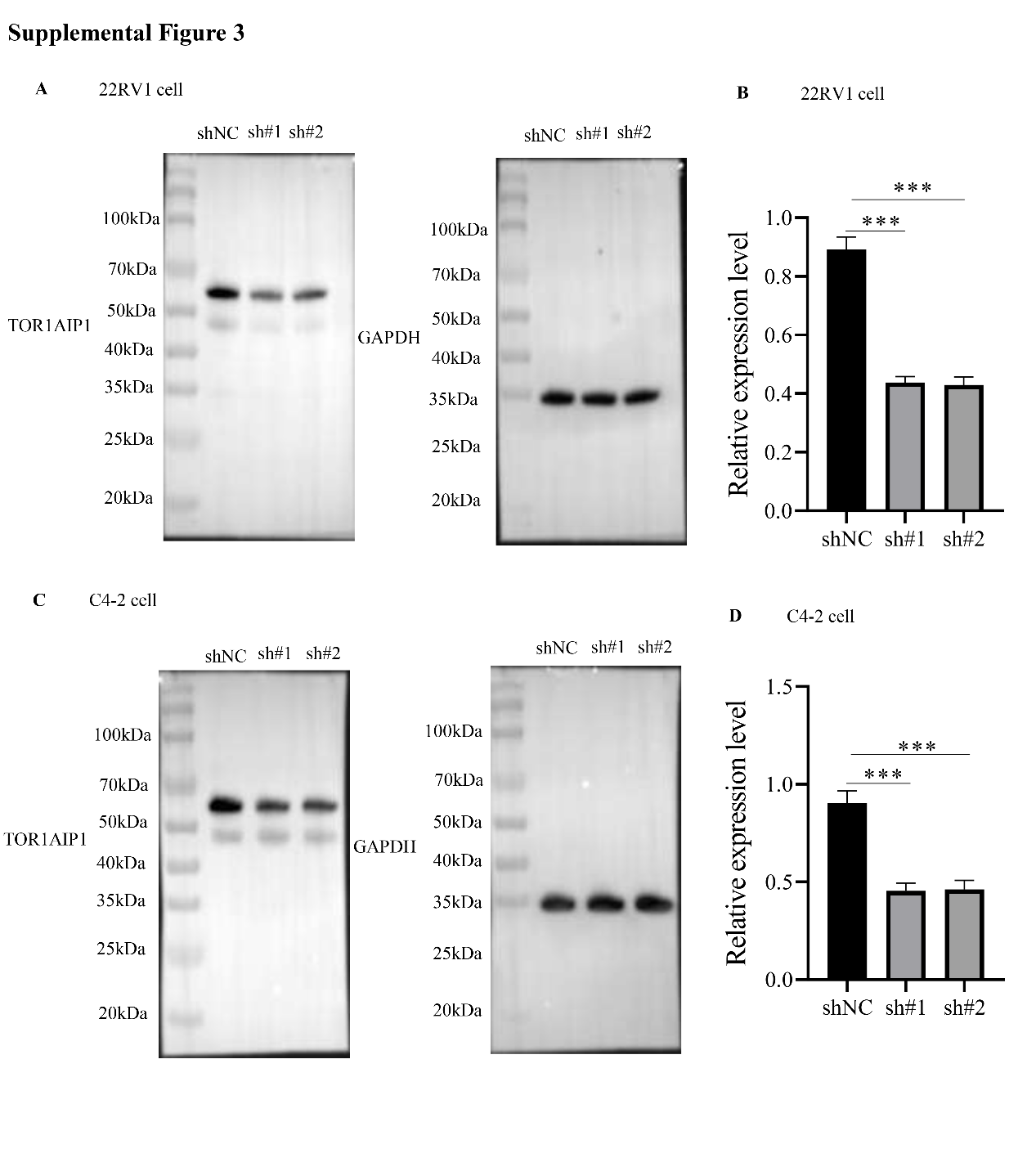


**The knockdown efficacy of TOR1AIP1. (A-D)** The knockdown efficacy of TOR1AIP1was determined by WB.

**Figure S5**


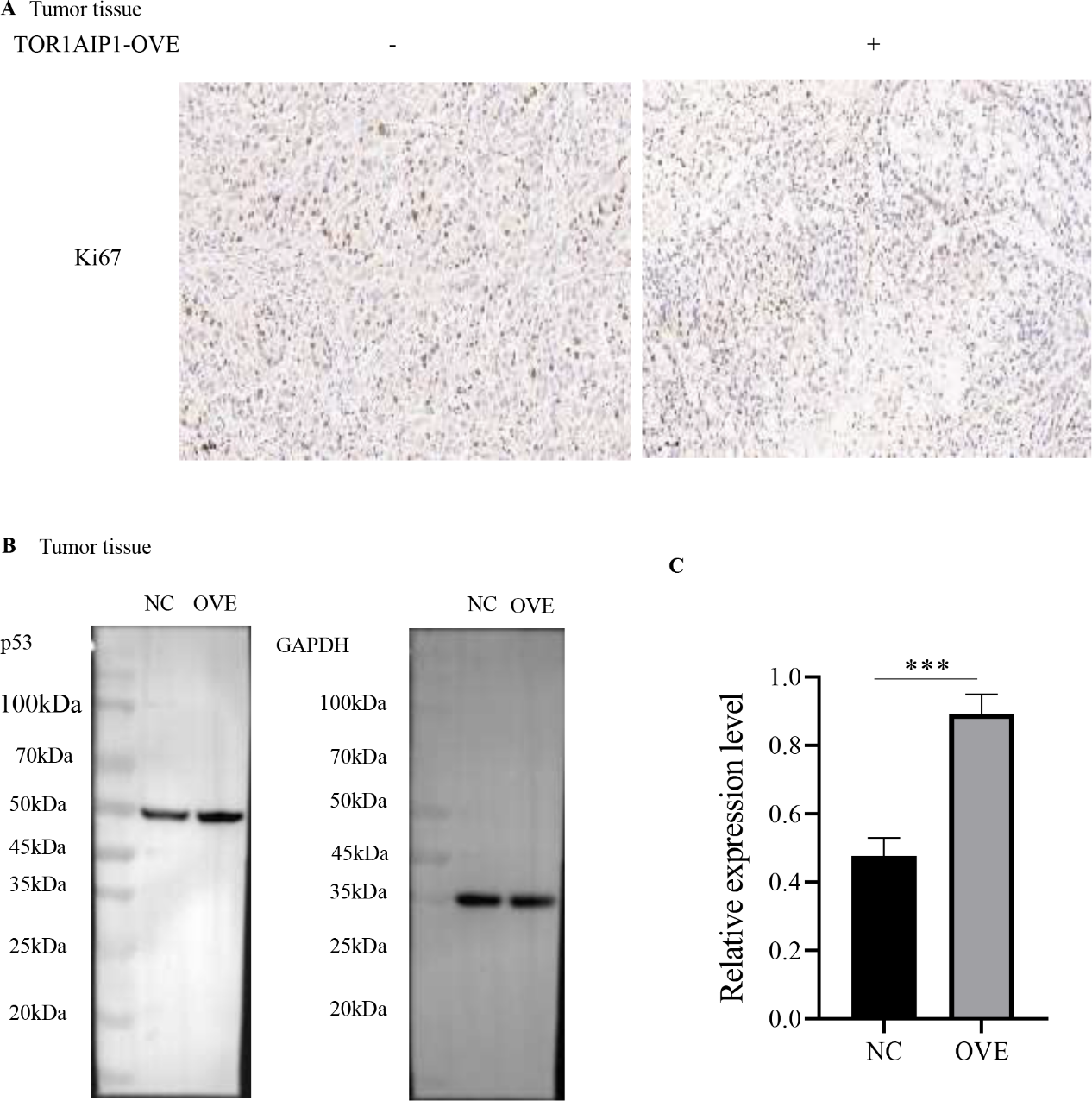


**The expression of proliferative markers ki67 and p53 protein in mouse tumor tissue. (A)** The expression of proliferation markers ki67 was determined by immunohistochemical. **(B-C)** The expression of p53 protein was determined by WB.

**Figure S6**


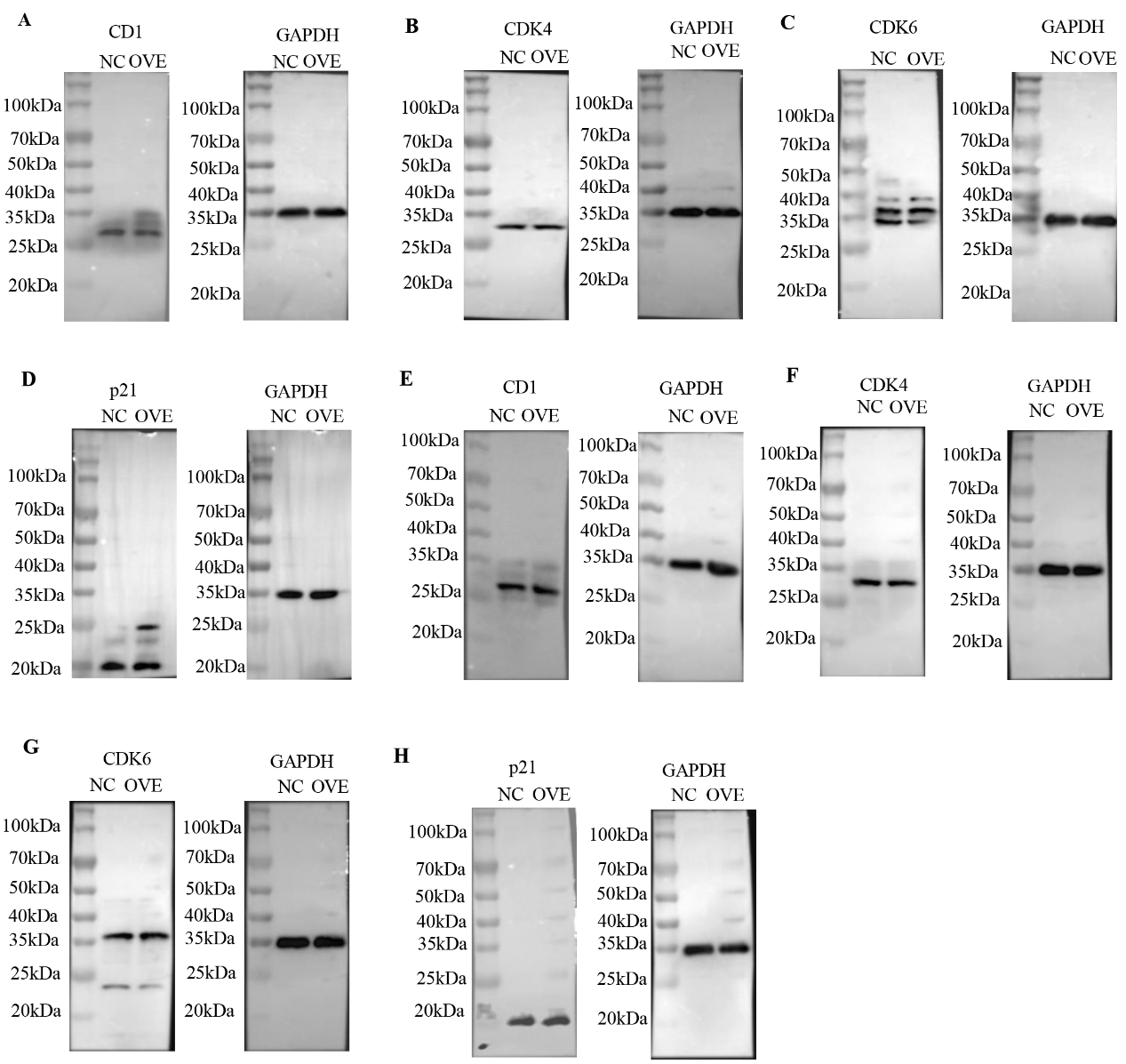


**The expression of CD1, CDK4, CDK6, p21 protein in DU145 and PC3M cells. (A-D)** The expression of CD1, CDK4, CDK6, p21 protein in DU145 cells. **(E-H)** The expression of CD1, CDK4, CDK6, p21 protein in PC3M cells.

**Figure S7**


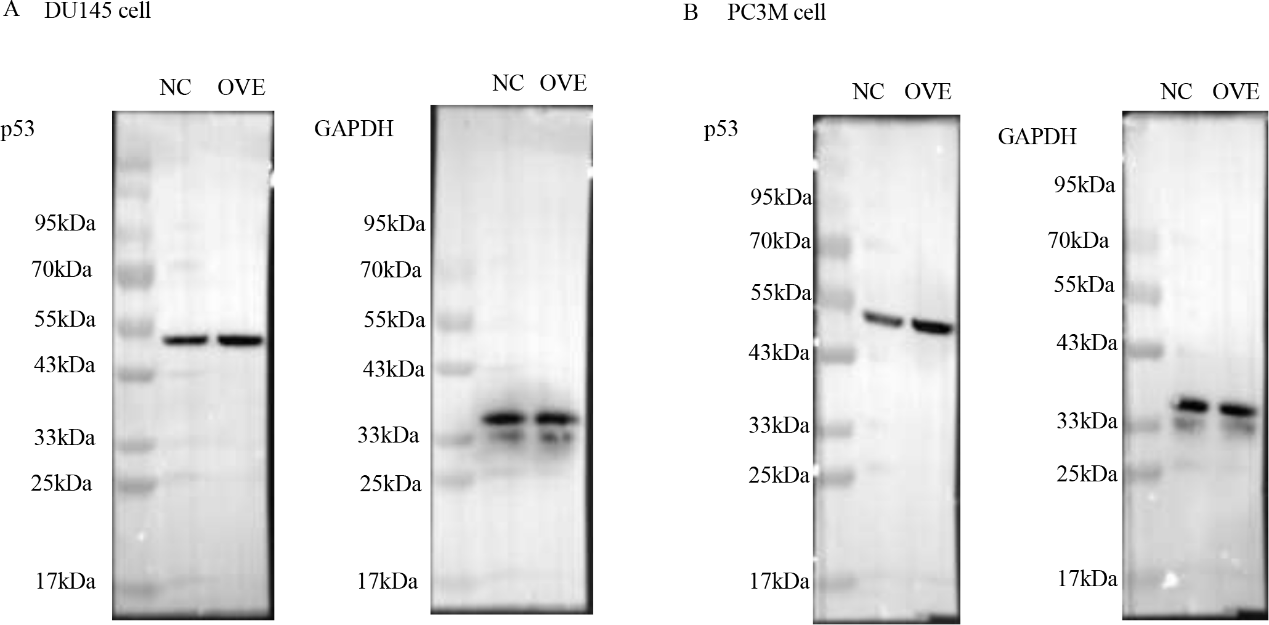


**Raw data of western blot for Fig 6D.**

**Figure S8**


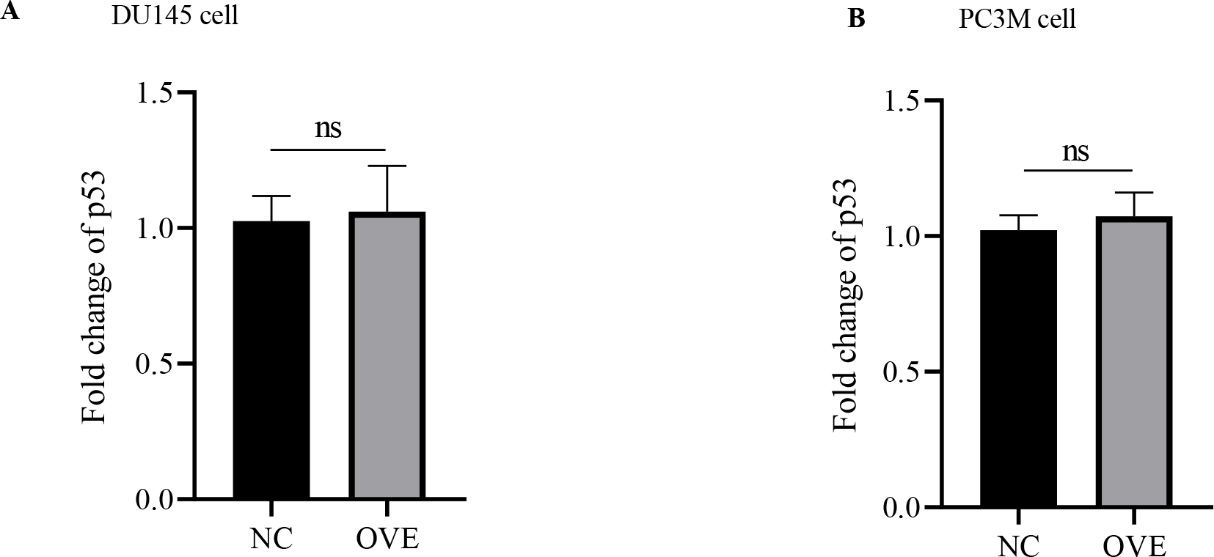


**The mRNA expression level of p53 in DU145 and PC3M cells. (A)** The mRNA expression level of p53 in DU145 cell. **(B)** The mRNA expression level of p53 in PC3M cell.

**Figure S9**


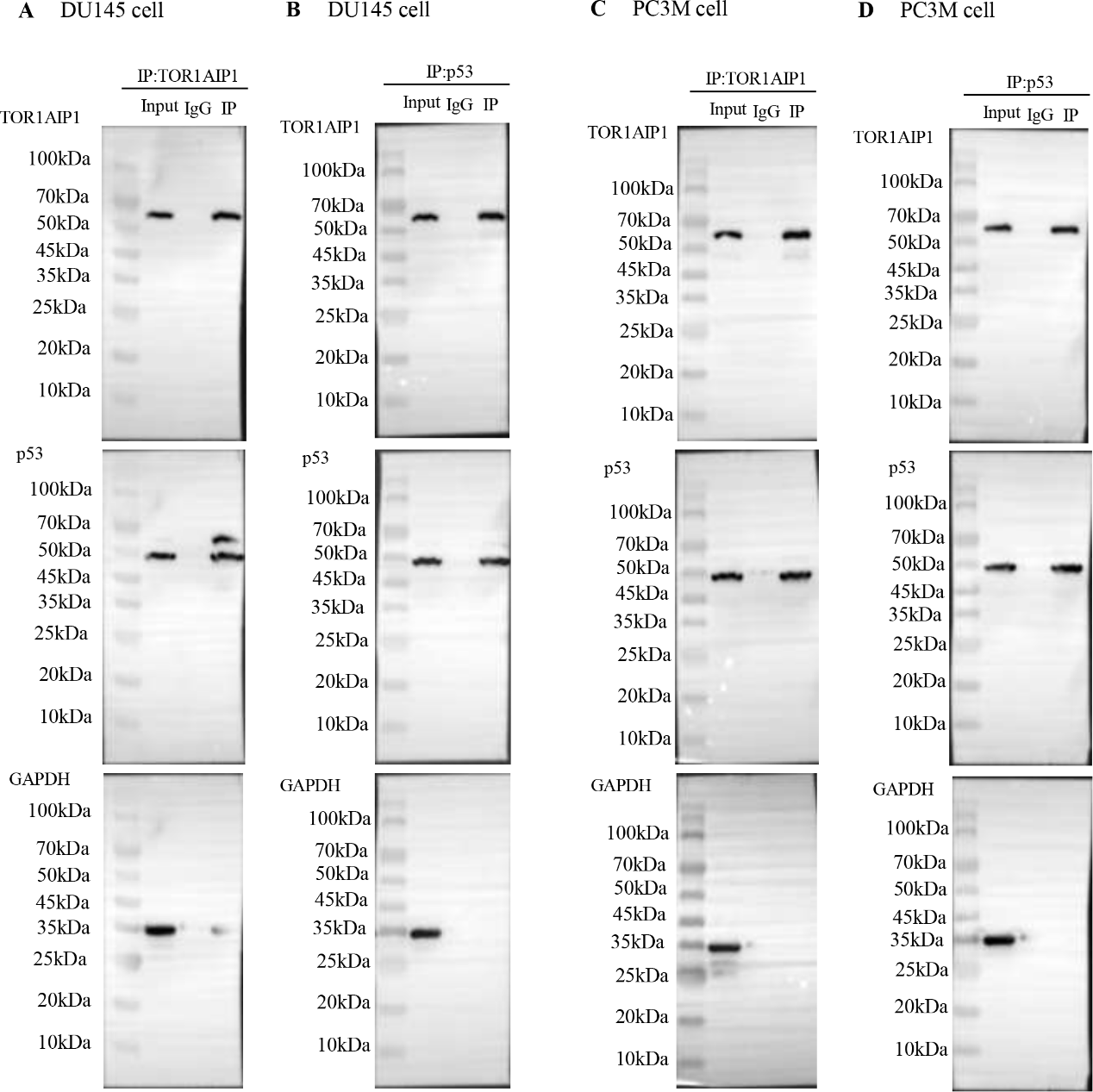


**Raw data of western blot for Fig 6F and 6G. (A-B)** Raw data of Fig 6F. **(C-D)** Raw data of Fig 6G.
